# Anti-inflammatory activity of *Jatropha curcas* Linn. latex in cream formulation on CD68 expression in mice skin wound

**DOI:** 10.14202/vetworld.2018.99-103

**Published:** 2018-02-02

**Authors:** Muhammad Nur Salim, Dian Masyitha, Abdul Harris, Ummu Balqis, Cut Dahlia Iskandar, Muhammad Hambal, Darmawi

**Affiliations:** 1 Laboratory of Pathology, Faculty of Veterinary Medicine, Syiah Kuala University, Banda Aceh 23111, Indonesia; 2 Laboratory of Histology, Faculty of Veterinary Medicine, Syiah Kuala University, Banda Aceh 23111, Indonesia; 3 Laboratory of Pharmacology, Faculty of Veterinary Medicine, Syiah Kuala University, Banda Aceh 23111, Indonesia; 4 Laboratory of Parasitology, Faculty of Veterinary Medicine, Syiah Kuala University, Banda Aceh 23111, Indonesia; 5 Laboratory of Microbiology, Faculty of Veterinary Medicine, Syiah Kuala University, Banda Aceh 23111, Indonesia; 6 Laboratory of Research, Faculty of Veterinary Medicine, Syiah Kuala University, Darussalam, Banda Aceh 23111, Indonesia

**Keywords:** anti-inflammatory, CD68, *Jatropha curcas* latex cream, wound healing

## Abstract

**Aim:**

The purpose of the present study was to determine the potential of *Jatropha curcas* latex in the cream formulation on CD68 immune expression (macrophages) during inflammatory phase wound healing process in mice skin.

**Materials and Methods:**

Amount of 12 two-months-old male mice were used between 30 and 40 g. To surgical procedures, wound skin incision was performed 2.0 cm in length until subcutaneous on the paravertebral of each animal. The treatment was carried under locally anesthetized with procaine cream. The mice were allotted into four groups of each, entire surface of each group wound covered by base cream control, sulfadiazine 0.1% cream, *J. curcas* latex cream 10% and, 15%, respectively. All experiments were performed twice a day for 3 days. The wound healing was assayed in stained histological sections in immunohistochemical of the wounds. CD68 expression was investigated under a microscope.

**Results:**

The results showed that the cream from the 10% and 15% latex of *J. curcas* revealed moderate immune reaction to CD68 on wound healing.

**Conclusion:**

We concluded that the latex cream of *J. curcas* possesses anti-inflammatory activity in wound healing process of mice skin.

## Introduction

On the basis of the physiology of wound healing, the wounds can be classified as chronic and acute wounds. Balqis *et al*. [1] documented that chronic wound appeared in an 18-years-old male *Elephas maximus* sumatranus due to surgical debridement to remove tumor extraskeletal fibrosarcoma in the captive elephant. Acute wounds caused by accidental tripping have been found in wild animals such as tiger, *Panthera tigris* sumatrae [2]. Indeed, these wounds contribute to substantial morbidities such as increased risk for infection, limb amputation, and animal death. It is understood that there are certain essential drugs present in modern medicine that help in the healing of wounds. There are several growth factors which are having the potential of improving the healing of wounds. Furthermore, a number of antibiotic compounds have been used in the treatment of the bacterial infected wounds. Unfortunately, there is a serious untoward effect such as carcinogenesis can be generated by some of these growth factors [3,4]. Using commercial antibiotic compounds leads to the risk of widespread development of resistant bacteria to most of the current antibiotics. A number of antibiotics resistant can be demonstrated in *Staphylococcus aureus* bacteria isolated from a human in Mongolia [5], from milk in South Africa [6], and from poultry in Malaysia [7]. Multidrug resistance occurred in *Escherichia coli* strain [8] and *Salmonella* spp. [9]. Due to the risks, various authors made a new path to introduce alternative wound healing agents from natural origin. The process of wound healing occurs in three phases such as inflammatory, proliferative, and maturation [10]. Importantly, the inflammatory phase is naturally intended to remove devitalized tissue and prevent invasive infection [11]. The macrophages are prominent inflammatory cells in wounds. Macrophages are phagocytes, contributing to both innate immunity and cell-mediated immunity. Their function is to stimulate lymphocytes and other immune cells to respond to the pathogens and to phagocytize cellular debris and pathogens [12,13]. The CD68 protein belongs to a family of the lyso­somal glycoprotein which is specifically expressed by tissue macrophages including Langerhans cells. Macrophages have many functions in wound healing, including host defense, the promotion and resolution of inflammation, the removal of apoptotic cells, and support of cell proliferation and tissue restoration following injury [14].

Due to its availability, small size rodent, easy of handling and low cost, mice are used as suitable animals model for the study of skin wound healing that closely parallels to the healing of human wounds. Various authors choose mice to carry out their study. For wound healing and antimicrobial properties, a study employed by Agra *et al*. [15] showed that the aqueous extract from *Bowdichia virgilioides* stem barks can be used in mice. Jiao *et al*. [16] described that the function of cell-mediated immunity of mice was influenced by flavonoid extracted from stem and leaf of *Astragalus membranaceus*. Uche and Aprioku [17] demonstrated that the analgesic and anti-inflammatory effects of methanol extract of *Jatropha curcas* leaves in mice. Previously, Mujumdar and Misar [18] used the mice as animals’ model in the study of the anti-inflammatory activity of *J. curcas* roots. In another study using leaf extract; Salim *et al*. [19] noticed that the efficacy of *J. curcas* Linn. on the process of wound healing in mice.

*J. curcas* Linn. from the Euphorbiaceae family has been used in many parts of the world for various medicinal purposes [20]. The leaf and latex extracts of *J. curcas* contained appreciable amounts of secondary metabolic compounds [21-23]. Leaves of this plant reported possessing antivirus on human immunodeficiency virus activity [24]. Extracts from this plant have been reported to have remarkable anti-inflammatory and antibacterial [25], cosmetic [26] and wound healing [27,28] hemostatic [29], antioxidant, and anticancer [30] potential. Unfortunately, the effect of this medication during the inflammatory phase of wounds healing process has not been fully defined.The present study was, therefore, conducted to evaluate *J. curcas* latex activity in cream formulation on CD68 immune expression (macrophages) during the inflammatory phase of wound healing process in mice skin.

## Materials and Methods

### Ethical approval

All experimental animal procedures were performed in compliance with the regulation of Animal Ethics Committee. This research was approved by the Animal Ethics Committee of Faculty of Veterinary Medicine, Syiah Kuala University, Banda Aceh, Indonesia (Approval No. 004/KEPH-C/VII/2017).

### Experimental animals

Amount of 12 male mice (*Mus musculus*) 2 months old and weighing 30-40 g obtained from Faculty of Veterinary Medicine, Syiah Kuala University, Banda Aceh, Indonesia, were used in current study. The mice were housed in individual cages which include drinking water and rations. The mice were fed a standard laboratory diet and given *ad libitum* access to food and water. The animals were kept for acclimatization for 2 weeks.

### Preparation of cream

The latex of *J. curcas* was obtained from a local farm in Darussalam village, Banda Aceh City, Aceh Province, Indonesia. The latex was collected in the morning with a break of leaf stems, latex at capacity into a sterile bottle. *J. curcas* latex cream made with a base of oil in water (O/W) according to methods Muntiaha *et al*. [31]. A cream base was added little by little with the 10% and 15% latex of *J. curcas* in a porcelain dish containing 100 g of cream and stirred until homogeneous at room temperature.

### Surgical procedures

All mice were anesthetized locally with procaine cream, and one wound skin incision was performed 2.0 cm in length until subcutaneous on the paravertebral of each animal. The animals were handled in accordance with aseptic principles to avoid exogenous bacterial contamination.

### Experimental design

Mice were divided into four groups with three mice of each. Group 1(A) as a negative control, the entire surface of wound covered by base cream. Group 2(B) as a positive control, the entire surface of wound covered by sulfadiazine 0.1% cream both Group 3(C) and Group 4(D) as treatment groups. The C and D, the entire surface of wound covered by *J. curcas* latex cream 10% and 15%, respectively. The wound was treated twice a day at 08.00 am and 18.00 pm until day 3.

### Histopathological and immunohistochemical expression of CD68

Wound skin tissue samples were collected in 10% buffered formalin for histopathological examination [32,33]. The tissues were processed by routine paraffin embedding technique, and 5 µm sections were stained with immunohistochemical staining by standard methods as described by Darmawi *et al*. [34], using streptavidin-biotin complex (SB). For macrophages immunoreaction, we used the CD68 monoclonal mouse antibody (Dako, Carpentaria, CA) at dilution 1:100 in PBS. Immunohistochemical labeling for each antibody was graded on scale of 0-3 Grades according to the following assessment: No detectable labeling (Grade 0), weak or mild labeling (Grade 1), moderate labeling (Grade 2), marked labeling (Grade 3) with local, and/or widespread reactivity as described by Caffo *et al*. [35].

## Results

**Figure-1:**
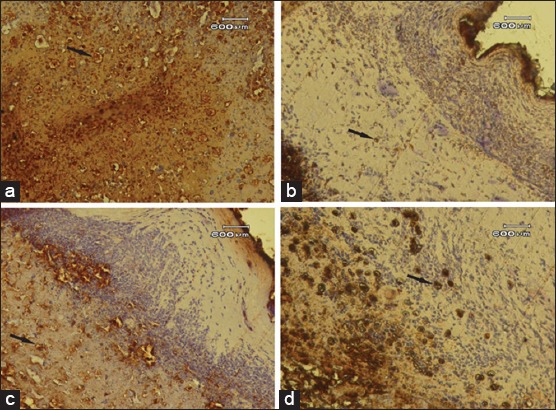
Photomicrograph of wounds skin on day 3 of treatment (streptavidin-biotin 40×). CD68 positive cells each group that marked the brown color (arrow). (a) Minimal immunoreactivity CD68 positive cells on Group A (Grade 1). (b-d) Moderate immunoreactivity CD68 positive cells in Groups B-D (Grade 2).

The results of the present study showed that the cream from the 10% and 15% latex of *J. curcas* revealed moderate immune reaction to CD68 on wound healing. The CD68 positive cells were especially detected in perivascular area. The CD68 monoclonal mouse antibody had a strong reaction with antigen on the connective tissue macrophages as seen in Figure-1. On day 3 wound healing process mice skin, the expression of CD68 positively reacts in four group treatment. Immunohistochemical examination with SB staining revealed macrophages in all the organs examined. The expression of macrophages in the skin tissue can be identified by the expression of the CD68 marker for a positive result. CD68 positive cells closed to perivascular area. Macrophages were identified as brown color against a pale ground background. Here, we regarded them as Langerhans macrophages.

Our result showed that the mice wound skins covered by base cream had a mild immune reaction to CD68 expression. We found that dramatic change of mice wounds skins in the appearance of CD68 expression treated with sulfadiazine 0.1%. We explained that dramatic changes of mice wound skins were observed in most of treated with *J. curcas* latex cream groups. Comparatively, the concentration of *J. curcas* latex cream 10% showed less effect than *J. curcas* latex cream 15%. Treatment of mice wounds skins with *J. curcas* latex cream 15% resulted in dramatic change in moderate immune reaction to CD68 expression as seen in Figure-1.

## Discussion

The simplest interpretation of our finding is that at least some macrophages go through the wound skins. In the study of Guo *et al*. [36] showed that immunohistochemical examination CD68 macrophages can determine in the process of wounds healing are more specific than histopathological with routine staining. Macrophages on the tissue can be identified by marker human CD68. Nucera *et al*. [37] described that CD68 was a 110kDa transmembrane glycoprotein by human monocytes and macrophages. CD68 can be used for identifying a population of cells being of mononuclear phagocyte origin, assessing the number of macrophages infiltrating a wound healing area. CD 68 expressed by Langerhans cells constitute, the most abundant macrophage population in the skin was observed as a major gateway for wound infection. In another study showed that the majority of macrophages found in the perivascular area of wound healing [28]. In this study, *J. curcas* latex cream 15% is an optimum dose that could fasten the inflammatory phase wound healing process. It was found that the process of wound healing in D is better than the other groups.

The present study clearly demonstrated that the CD68 expression on mice wound skins it can be seen that the *J. curcas* latex cream 15% have potential as an anti-inflammatory. In support of this hypothesis, many previous reports exist about the effect of using *J. curcas* as therapeutic agents that contributes to impaired wound healing process. These extracts showed good antioxidant [22], coagulant and anticoagulant [38], analgesic, antibacterial, and anti-inflammatory [25]. A similar outcome was observed the effect of n-hexane *J. curcas* leaf extract showing the hemostatic effect that exhibited a significant decreased in bleeding time [29]. The latex from branches has been used in wound healing, refractory ulcers, and septic gums and as a styptic in cuts and bruises [28]. These findings are not different from those reported the effect of *J. curcas* extract revealed that reduction of bleeding time increased proportionally with the increase in extract concentration [29]. Regarding the activity of *J. curcas* extract to impaired wound healing process similar for and support those of Shetty *et al*. [27], who observed in albino rats showed that extract of *J. curcas* bark was potential in accelerating wound healing.

There are some secondary metabolic compounds extracted from *J. curcas* involved in wound healing process. Abdelgadir and Staden [20] described that the leaf and latex extracts of *J. curcas* contained appreciable amounts of phenolic and saponin compounds that are responsible for antimicrobial and antioxidant activities. Importantly, Esimone *et al*. [28] explained that flavonoid quercetin and rutin can improve wound healing process in the initial phase, namely, the regulation of the expression of vascular endothelial growth factor (VEGF) for the growth of new blood vessel and formation of collagen Type III. However, flavonoid compounds play a role in the early phase of wound healing (inflammatory phase) to increase the activity of the immune system of the body such as interleukin-2, proliferation of lymphocytes, and macrophages [16]. Moreover, sesquiterpenoids are responsible for antimicrobial and analgesic effects, meanwhile, proteins such as curcain are responsible for wound healing [20]. Therefore, Jatropha oil has anti-inflammatory activity [17,18,39]. The leaves and latex of *J. curcas* contained phenolic compounds and saponins that have antioxidant and anti-inflammatory activity [40]. Flavonoid as an antioxidant can reduce free radicals and will bond with free radical that damage cell membranes [41]. Flavonoids accumulated in the latex of *J. curcas* play an essential role in inflammatory phase by increasing interleukin-2, proliferation of lymphocytes, and macrophages [16]. Indeed, saponins can increase the proliferation of monocytes which eventually will increase the number of macrophages that secrete the growth factor which crucial for wound healing process [42].

The evidence described here argues that the *J. curcas* latex cream is also possibly involved in the early phase of wound healing. In confirmation of our previous study, we found that *J. curcas* latex cream histopathologically could be decreased inflammatory cells infiltration [19]. Various authors noticed that the purpose of inflammation was interesting to plasma protein and phagocytic cells to the wound surface to destroy foreign substances, cell debris, and prepared for the process of wound healing and repair [36,37,43,44]. In respect of our results, the finding is similar to those described by Singer and Clark [43], so it seems macrophages were widely distributed in the body and played a role in the process inflammation as the body’s reaction to foreign particles and bacteria. It has long been known that the macrophages play an important role in inducing angiogenesis in a manner secrete several factors: Tumor necrosis factor-alpha, VEGF, angiogenin, urokinase, and platelet-derived growth factor. The role of macrophages CD68 on angiogenesis (CD34 marker) in line with research conducted by El-Rouby [44] showed that the number of macrophages, which was widely followed by an increasing amount of angiogenesis.

## Conclusion

We concluded that the latex cream of *J. curcas* possesses anti-inflammatory activity in wound healing process of mice skin.

## Authors’ Contributions

MNS and AH designed and carried out the main research work. The research was carried by DM, UB, and CDI. The manuscript was written by MNS, Darmawi, UB, CDI, and MH. All authors read and approved the final manuscript.
